# Quantification of Head Acceleration Events in Rugby League: An Instrumented Mouthguard and Video Analysis Pilot Study

**DOI:** 10.3390/s22020584

**Published:** 2022-01-13

**Authors:** James Tooby, Dan Weaving, Marwan Al-Dawoud, Gregory Tierney

**Affiliations:** 1 School of Biomedical Sciences, University of Leeds, Leeds LS2 9JT, UK; 2 Carnegie Applied Rugby Research (CARR) Centre, Carnegie School of Sport, Leeds Beckett University, Leeds LS1 3HE, UK; d.a.weaving@leedsbeckett.ac.uk (D.W.); G.Tierney@ulster.ac.uk (G.T.); 3 Leeds Rhinos Rugby League Club, Leeds LS5 3BW, UK; marwan.al-dawoud@leedsrugby.com; 4 Sport and Exercise Sciences Research Institute, School of Sport, Faculty of Life and Health Sciences, Ulster University, Belfast BT15 1ED, UK

**Keywords:** biomechanics, head impact, concussion, subconcussion, kinematics

## Abstract

Instrumented mouthguards (iMG) were used to collect head acceleration events (HAE) in men’s professional rugby league matches. Peak linear acceleration (PLA), peak angular acceleration (PAA) and peak change in angular velocity (ΔPAV) were collected using custom-fit iMG set with a 5 g single iMG-axis recording threshold. iMG were fitted to ten male Super League players for thirty-one player matches. Video analysis was conducted on HAE to identify the contact event; impacted player; tackle stage and head loading type. A total of 1622 video-verified HAE were recorded. Approximately three-quarters of HAE (75.7%) occurred below 10 g. Most (98.2%) HAE occurred during tackles (59.3% to tackler; 40.7% to ball carrier) and the initial collision stage of the tackle (43.9%). The initial collision stage resulted in significantly greater PAA and ΔPAV than secondary contact and play the ball tackle stages (*p* < 0.001). Indirect HAE accounted for 29.8% of HAE and resulted in significantly greater ΔPAV (*p* < 0.001) than direct HAE, but significantly lower PLA (*p* < 0.001). Almost all HAE were sustained in the tackle, with the majority occurring during the initial collision stage, making it an area of focus for the development of player protection strategies for both ball carriers and tacklers. League-wide and community-level implementation of iMG could enable a greater understanding of head acceleration exposure between playing positions, cohorts, and levels of play.

## 1. Introduction

Rugby league is a contact sport played around the world at different levels. Due to the high number of collisions that players are involved in [1], there is a risk of both musculoskeletal and concussion injuries [2]. Concussion injuries and head acceleration exposure in rugby are a concern owing to the potential long-term consequences [3], but have yet to be fully investigated.

Within the European Super League, the incidence of concussion has increased from two and three concussions per 1000 player-hours in 2013 and 2014 to eight concussions per 1000 player-hours in 2015 [4]. In the Australian National Rugby League, the concussion rate was reported at 15 concussions per 1000 player-hours during the 2013 season [5]. The mechanism of concussion in rugby league has been investigated through video analysis in previous studies [5,6,7]. Some evidence suggests that the ball carrier is at a greater risk of concussion than the tackler [6]. However, another study found that tacklers were removed from play using the concussion interchange rule at a greater rate than ball carriers [7].

Head acceleration events (HAE) can be caused by direct head impacts (direct HAE) or inertial head loading from body contact (indirect HAE) [8]. High-magnitude HAE are associated with a risk of concussion [9]; however, a global injury tolerance remains unknown [10]. HAE that do not result in acute concussion could be considered synonymous to sub-concussive impacts. Recent evidence suggests that increased exposure to repetitive HAE may reduce an individual’s tolerance for concussion [11] and has been proposed as a secondary mechanism of concussion [12]. This demonstrates the need to monitor head acceleration exposure during training and matches. Quantifying the accumulation of HAE over time may be a better predictor than concussion history for predicting long-term clinical outcomes such as cognitive impairment, depression, and executive dysfunction in former athletes [13]. However, more longitudinal studies are needed to further understand the extent of these relationships. A proactive approach to player protection would be to develop strategies to reduce the magnitudes of HAE from impacts to the head and body without compromising the dynamics of the game, such as rule changes or coaching techniques. In order to develop player protection strategies, areas of the sport need to be identified where HAE occur most commonly and with the highest magnitude.

HAE kinematics can be measured using wearable head sensors instrumented with accelerometers and/or gyroscopes [14]. Instrumented patch devices suffer from poor skull coupling that create a tendency to overestimate HAE magnitude [14]. Instrumented mouthguards (iMG) have been demonstrated to show superior coupling with the skull through the upper dentition and are recommended for in vivo measurement of HAE [14,15]. iMG have been used to measure HAE in rugby union [15,16] and American Football [8], but not in rugby league. The combination of qualitative video analysis with objective kinematic measures can allow us to identify the contact events which lead to the highest magnitude and frequency of HAE [8,15,17]. The aim of this study was to identify and quantify HAE sustained during competitive men’s professional rugby league matches based on tackle stage, head loading type and player role.

## 2. Materials and Methods

Ten male players were recruited from a Super League team including two backs (centres) and eight forwards (two props, two second rows, two loose forwards, a hooker and a back row). Each player provided written consent and ethical approval was given for this study by the Faculty of Biological Sciences Ethical Review Committee, University of Leeds (#BIOSCI 18-023). The mean age of the players was 20.1 (±3.3) years, the mean weight was 99.0 kg (±8.7) kg, and the mean height was 183.8 (±7.3) cm. Data were collected for 1596 active player minutes over 31 player matches in the 2019 season. Active player minutes were calculated for each player as the amount of time from either the start of the half or the player entering the pitch after an interchange, up until either the end of the half or the player leaving the pitch after an interchange. In matches where the iMG were improperly charged and stopped recording HAE before the end of the match (*n* = 3), active minutes were calculated up until the final recorded HAE. 

A custom-fit iMG (Prevent Biometrics, Minneapolis MN) was utilised in this study, which is fitted with an accelerometer and gyroscope both sampling at 3200 Hz, with measurement ranges of ±200 g and ±35 rad/s, respectively. An infrared proximity sensor embedded in the iMG was utilised to assess if the iMG was coupled tightly to the upper dentition during HAE. The reliability and validity of the Prevent Biometrics custom-fit iMG has been demonstrated in previous studies [15,18,19]. Kieffer et al. [19] conducted a two-phased approach to assess the accuracy of a range of wearable head sensors. The first phase consisted of laboratory-based impact testing on a crash test dummy headform and the second phase assessed if the sensors can detect true-positive HAE on the field. The highest performing device was the Prevent Biometric custom-fit iMG for both phases, with a concordance correlation coefficient value of 0.97 and a positive predictive value of 96% for active minutes (i.e., not including periods of inactivity such as substitutions and half-time). Similarly, Liu et al. [18] conducted laboratory-based impact testing on a crash test dummy headform to assess the accuracy of a range of iMG. The Prevent Biometric custom-fit iMG was identified as the best performing device with a mean relative error of 4.9%, 4.6% and 2.5% for peak angular acceleration, angular velocity, and linear acceleration, respectively. During pre-season, each athlete had digital dental impressions taken and custom-made iMG manufactured to ensure a tight fit to the upper dentition. 

The Prevent Biometrics custom-fit iMG recorded HAE kinematics when accelerometer measures exceeded 5 g on a single axis of the iMG, capturing 10 ms of pre-trigger data and 40 ms of post-trigger data. The level of noise was classified by an in-house Prevent Biometrics machine learning model which determined whether each HAE contained minimal noise (class 0), moderate noise (class 1) or severe noise (class 2). A 4-pole, zero phase, low-pass Butterworth filter was applied at cut-off frequencies of 200 Hz for class 0 HAE (*n* = 1700), 100 Hz for class 1 HAE (*n* = 60) and 50 Hz for class 2 HAE (*n* = 23). In-house Prevent Biometrics algorithms transformed linear kinematics to the head centre of gravity (CG). An initial HAE verification analysis was conducted by Prevent Biometrics using video verification and infrared proximity and light sensor values to develop their HAE impact detection algorithm. Only HAE determined to be true positives as per the HAE impact detection algorithm were considered in the analysis (*n* = 1783). 

**Figure 1:**
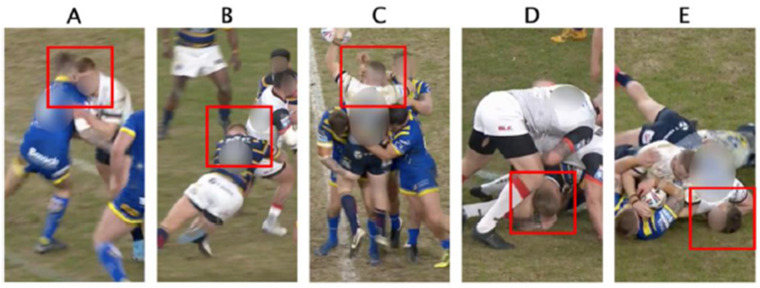
HAE labelled as Initial Collision for a (**A**) carry, (**B**) tackle; (**C**) Secondary Contact, (**D**) Ground Contact and (**E**) Play the Ball for tackle stage, see Table 1 for definitions. An initial tack-le/carry contact between the two impacting players had already been made prior to the arm-to-head HAE pictured in the (**C**) Secondary Contact. Red squares indicate the impacted player.

Peak linear acceleration (PLA) and peak angular acceleration (PAA) were extracted from the resultant values fitted to the head CG. Peak change in angular velocity (ΔPAV) was calculated for each HAE by extracting the peak value from the post-trigger angular velocity components that were zeroed to the 5 g single axis trigger point (10 ms). ΔPAV was preferred to PAV to measure the change in angular velocity as a result of the impact [15]. Broadcast quality video footage was available for all matches and iMG timestamps from each HAE were used to synchronise HAE to video footage to a 40 ms resolution enabling video verification and qualitative analysis. HAE with timestamps outside of the video footage were not included in the analysis (*n* = 68). Qualitative video analysis was conducted on all true-positive HAE to determine the type of contact event, tackle stage (Figure 1), impacted player, and head loading type (Table 1). The 6 degree-of-freedom head displacement during the HAE were reconstructed from the linear and angular kinematic time-series data using a customised MATLAB script to improve the raters’ ability to identify HAE in the video and head loading type. HAE that triggered immediately following another HAE without another visible contact on video (*n* = 44), HAE that had no corresponding contact event on video (*n* = 18), and HAE occurring when the video footage was not following open play, e.g., recording the crowd (*n* = 31) were all removed from the analysis. Any indistinguishable characteristics were labelled as occluded (head loading type: *n* = 15). Inter-rater reliability for each characteristic was assessed using Cohen’s κ [20] (Table 1).

### Statistical Analysis

All statistical analyses were conducted in R Studio using the lme4 package [21]. Dependent variables included continuous variables PLA, PAA and ΔPAV. Independent variables (i.e., fixed effects) included tackle stages (initial collision, secondary contact, ground contact and play the ball; and direct and indirect HAE) (Table 1), which were treated as categorical variables. Non-tackle HAE and HAE with characteristics labelled as occluded were not considered in the statistical analysis. Histograms and Q-Q plots were used to visually inspect data for normality. No data followed a normal distribution; therefore, all dependent variables were log-transformed prior to statistical analysis to reduce the error from non-uniform data. Nested data in clusters of individual players were observed, and so mixed-effects linear models were used [8]. PLA, PAA and ΔPAV were compared between tackle stages (model 1) and head loading types (model 2). For model 1, tackle stages were split based whether the impacted player was a ball carrier or tackler. In both models, the player was included as a random intercept. Multiple comparisons were accounted for by a Bonferroni correction. For each comparison, the effect size difference (95% confidence interval) was estimated from the ratio of the observed mean difference to the pooled standard deviation. Effect size (ES) differences were interpreted as trivial (<0.2), small (0.2 to <0.6), moderate (0.6 to <1.2), large (1.2 to <2) and very large (≥2) [8].

## 3. Results

**Figure 2:**
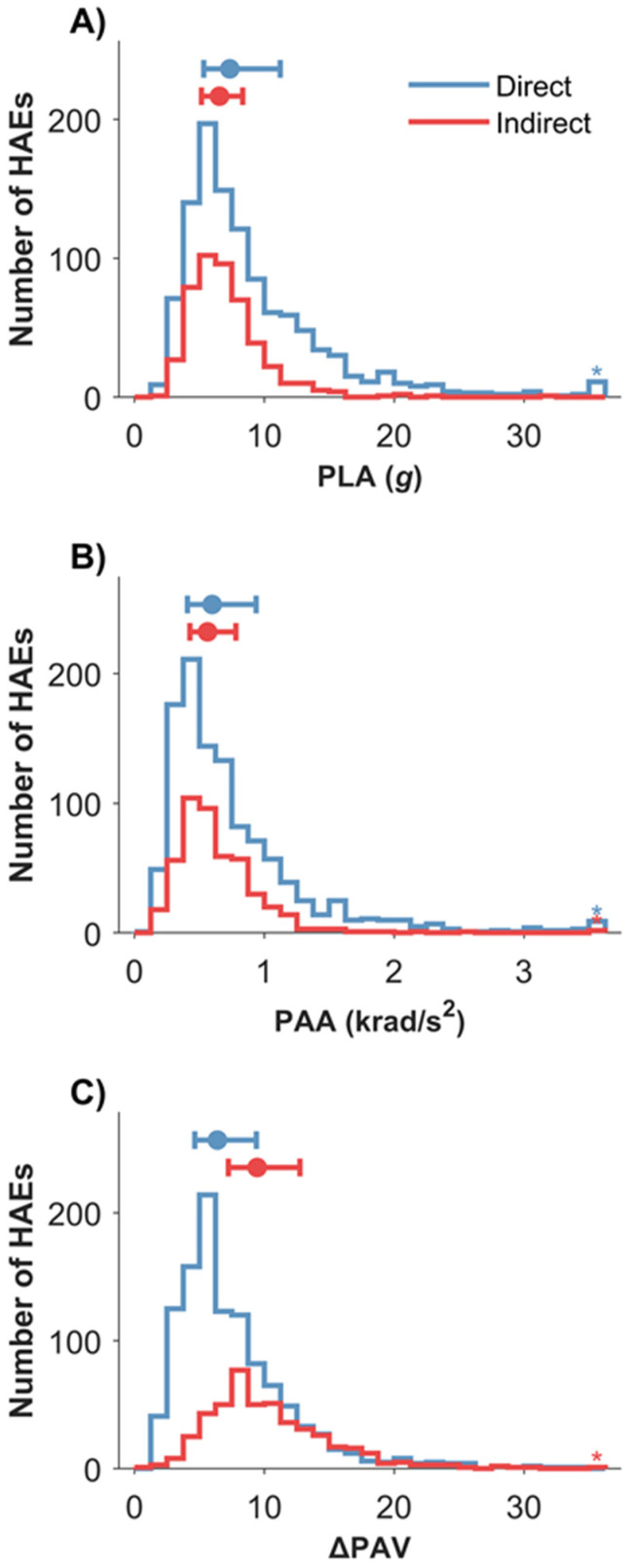
Distributions of direct (*n* = 1108) and indirect (*n* = 470) HAE, with plots showing the median and interquartile ranges for (**A**) PLA, (**B**) PAA and (**C**) ΔPAV. Asterisks indicate HAE above (**A**) 35 g (*n* = 10), (**B**) 3.5 krad/s^2^ (*n* = 11) and (**C**) 35 rad/s (*n* = 1) which can be seen in Figure 3.

In total, 1622 HAE from video-verified contact events were recorded. The distribution of HAE kinematics is shown in Figure 2. The median and interquartile range for PLA (median = 7.1 g, Q1 = 5.2 g, Q3 = 9.9 g), PAA (median = 0.6 krad/s^2^, Q1 = 0.4 krad/s^2^, Q3 = 0.9 krad/s^2^) and ΔPAV (median = 7.5 rad/s, Q1 = 5.1 rad/s, Q3 = 10.7 rad/s) can be seen in Figure 2. Approximately three-quarters (75.7%) of HAE from contact events were below 10 g, 91.2% below 15 g and 96.1% below 20 g.

Qualitative video analysis revealed that HAE sustained during a tackle comprised 98.2% of all HAE, with 59.3% of all HAE occurring to tacklers and 40.7% occurring to ball carriers. Non-tackle events were caused by the ball hitting the head (0.9%), head contact during celebrations (0.8%) and players contacting their own head (0.1%). 

**Figure 3:**
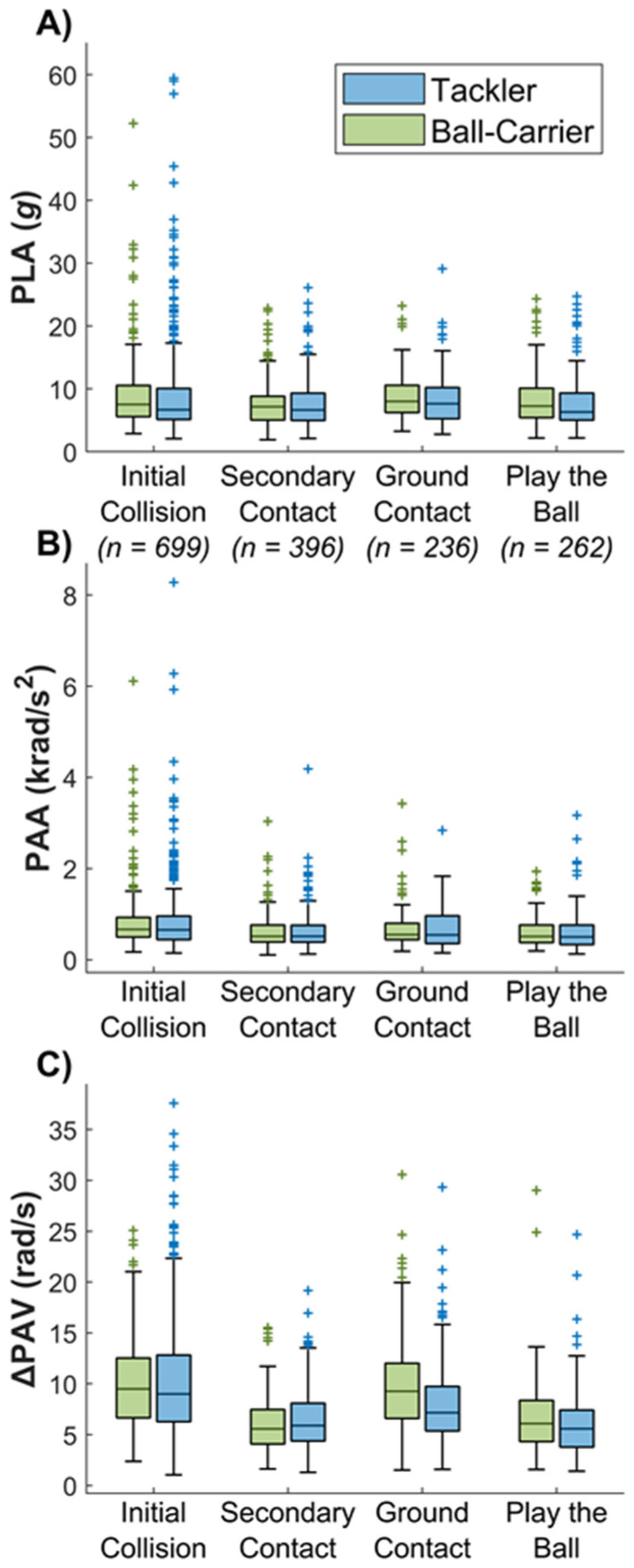
Box plots illustrating median and interquartile range (and outliers as crosses) (**A**) PLA, (**B**) PAA and (**C**) ΔPAV for different stages of a tackle: initial collision, secondary, ground contact and play the ball.

There were no significant differences in PLA or PAA between tacklers and ball carriers for any tackle stages (Table 2 and Figure 3). Ball carriers experienced significantly greater ΔPAV during HAE from ground contacts than tacklers (Table 2). Within tackle-related HAE, 43.9% occurred in the initial collision stage, 24.9% in the secondary contact stage, 14.8% occurred from ground contacts, and 16.4% occurred during the play the ball stage. PAA and ΔPAV were significantly greater from the initial collision stage than both the secondary contact and play the ball stages for both tacklers and ball carriers (Figure 3 and Table 2). Ground contacts also led to significantly greater ΔPAV than both secondary contact and play the ball stages for both ball carriers and tacklers. 

Direct HAE resulted in greater PLA than indirect HAE; conversely, indirect HAE resulted in greater ΔPAV (Figure 2 and Table 2). Direct HAE accounted for 70.2% of HAE, whilst indirect HAE accounted for 29.8% of HAE (Figure 2). For 95th percentile or greater HAE (PLA > 19.0 g or PAA > 1.7 krad/s^2^), 60.2% were from the initial collision stage and 93.2% were from direct head loading. The initial collision and ground contact stage accounted for 98.9% of all indirect HAE. For the ball carrier, indirect HAE accounted for 58.7% of HAE from the initial collision stage and 75.7% from the ground contact stage. For the tackler, indirect HAE accounted for 43.0% of HAE from the initial collision stage and 36.3% from the ground contact stage. 

## 4. Discussion

Qualitative video analysis revealed that most (98.2%) HAE occur during the tackle event. Given that the highest-magnitude HAE (95th percentile or above) for either PLA or PAA were caused by the initial collision stage (60.2%) and from direct head loading (93.2%), these HAE characteristics may represent the greatest scope for developing player protection strategies. HAE from the initial collision stage were significantly greater than HAE from the secondary contact and play the ball stages for both ΔPAV and PAA. However, to fully understand head acceleration exposure in rugby league, it is important to consider HAE that occur after the initial collision (e.g., secondary contact and ground contact). These HAE may not be detected by video analysis alone yet accounted for a considerable proportion of HAE in the present study and a similar instrumented ear patch study in junior rugby league [17]. Approximately one-quarter of HAE were indirect HAE, which led to significantly greater ΔPAV than direct HAE. Given that angular velocity has been demonstrated to correlate well with strain deformation in the brain [22], these results demonstrate the importance of including inertial head loading/body collisions when assessing head acceleration exposure in rugby league. Tierney et al. [8] found in a small sample of collegiate American football players that impacts labelled as indirect HAE by trained video reviewers resulted in greater head kinematics than direct HAE. However, the authors indicate that for the same given impact conditions, a direct HAE would result in greater head kinematics than an indi-rect HAE and that direct head contact likely occurred as a secondary impact mecha-nism during impacts labelled as indirect HAE. The findings indicate the short comings of using only qualitative video review when multiple HAE can occur during a single contact event. The inclusion of the customised MATLAB script that enabled 6 de-gree-of-freedom head displacement reconstructions in this study improved our ability to combat this issue.

Most studies utilising wearable head sensors in sport typically use a recording threshold of 10 g at the head CG [23]; however, 75.7% of HAE occurred below 10 g at the head CG in the current study. Accordingly, a robust comparison of our kinematic findings with other iMG studies is difficult. The clinical significance of the accumulation of these lower-magnitude HAE is still unknown. However, the omission of lower-magnitude HAE when a higher recording threshold is applied may withhold vital information on the loads placed on players’ brains over their careers [23]. Moreover, previous studies employing a 10 g threshold may underestimate head acceleration exposure rates experienced by contact sports players. Studies generally state that impact events that result in PLA < 10 g are possible from voluntary movements such as running and jumping [23], and thus are considered “non-contact events”. However, a recent systematic review [24] illustrates that head acceleration magnitudes during these types of “non-contact” events are dependent on the wearable head sensor used, with poorly coupled sensors (e.g., helmet and headband-based) typically showing higher magnitudes. A recent study illustrated that linear acceleration-based thresholds can lead to triggering biases that depend on sensor location and impact condition, with rotational acceleration-based thresholds (tentatively 700–750 rad/s^2^) considered more suitable [25]. Additionally, temporal data (e.g., time pulse and frequency content) of the kinematic signal may be necessary for distinguishing direct and indirect HAE from non-contact events. 

There were no significant differences in the magnitudes of head kinematics between ball carriers and tacklers for the initial collision, secondary contact and play the ball tackle stages, which supports that both ball carriers and tacklers need to be the focus of player protection strategies in rugby league. 

A comparison of the incidence between ball carrier and tackler HAE was not conducted in the present study due to the small and biased sample (more forwards than backs). The small sample size also meant that exposure rates and position specific data could not be reported. League-wide and community-level implementation of iMG could enable a greater understanding of head acceleration exposure between playing positions, cohorts and levels of play [26]. In this study, 4.7% of HAE were filtered at 50 or 100 Hz (class 1 and 2 HAE). The authors are unaware of a scientific justification for using lower cut-off frequencies for these “noisier” HAE. The signal processing requirements of class 1 and 2 HAE should be explored further, as these could be of higher magnitude than that reported by the iMG system. Additionally, the authors are unaware whether the signal attenuation is −3 dB or −6 dB at the class 0, 1 or 2 cut-off frequencies prescribed. The head kinematics used to describe HAE in this study (PLA, PAA and ΔPAV) also have limitations, such as ignoring directionality and temporal data (e.g., time pulse and frequency content) from the iMG kinematic signals, which may be important factors in predicting the degree of brain tissue deformation from a contact event [27,28,29]. The use of finite element brain model-based metrics may be more useful for describing HAE in future studies. Future work on the relationship between HAE kinematics and clinical outcomes such as concussion risk could potentially aid in the on-field decision-making process for sideline medical staff. Further research is needed to qualitatively analyse initial collisions from a tackle technique (e.g., tackle height and type) and player physical characteristic (e.g., height and weight) perspective to inform concussion prevention and HAE mitigation strategies.

## 5. Perspective

This study is the first to measure HAE kinematics in elite-level rugby league players using iMG. The majority of HAE from contact events are low in magnitude. Virtually all HAE occur during a tackle, with the initial collision accounting for more HAE than secondary contact, ground contact and play the ball stages. The initial collision also led to higher kinematics than other tackle stages, making it an area of focus for the development of player protection strategies for both ball carriers and tacklers. League-wide and community-level implementation of iMG could enable a greater understanding of head acceleration exposure between playing positions, cohorts and levels of play.

## Figures and Tables

**Table 1:** Qualitative Video Analysis Framework.

Characteristic	Cohen’s κ [20]	Definition
Contact Event	1	Tackle—HAE occurs whilst the tackler is attempting to impede the ball carrier Non-tackle—Any HAE occurring outside of a tackle (e.g., during celebrations, ball to head impacts).
Only tackle HAE were analysed for the following characteristics		
Tackle Stage	0.87	Initial Collision—HAE occurs from the first collision made between ball carrier and each unique tackler in a tackle. Secondary Contact—HAE occurs after the initial collision between the same ball carrier and tackler has already been made and before the ball carrier is grounded. Ground Contact—HAE caused by players falling to the ground during a tackle; forces can be transmitted through player. Play the Ball—Any HAE which occurs after the ball carrier has been grounded before a new phase begins.
Impacted Player	1	Ball Carrier—Impacted player is in possession of the ball. Tackler—Impacted player is attempting to stop the ball carrier.
Head Loading Type	0.82	Direct—Head loading is through direct contact with the head. Indirect—Inertial head loading transmitted through the neck from an impact to the body.

**Table 2:** Pairwise comparisons from mixed-effects linear models 1 and 2. Following a Bonferroni correction, the alpha value was set at 0.001. Significant comparisons are emboldened.

Pairwise Comparison	PLA		PAA		ΔPAV	
	ES	*p*-Value	ES	*p*-Value	ES	*p*-Value
	Ball Carrier HAE					
Initial collision vs. secondary contact	**0.3**	**0.003**	**0.5**	**<0.001**	**0.97**	**<0.001**
	(small)		**(small)**		**(moderate)**	
Initial collision vs. ground contact	−0.07	0.516	0.29	0.024	0.03	0.818
	(trivial)		(small)		(trivial)	
Initial collision vs. play the ball	0.14	0.202	**0.47**	**<0.001**	**0.79**	**<0.001**
	(trivial)		**(small)**		**(moderate)**	
Secondary contact vs. ground contact	−0.37	0.003	−0.21	0.131	**−0.95**	**<0.001**
	(small)		(small)		**(moderate)**	
Secondary contact vs. play the ball	−0.16	0.197	−0.04	0.792	−0.18	0.134
	(trivial)		(trivial)		(trivial)	
Ground contact vs. play the ball	0.21	0.106	0.18	0.245	**0.77**	**<0.001**
	(small)		(trivial)		**(moderate)**	
	**Tackler HAE**					
Initial collision vs. secondary contact	0.19	0.016	**0.41**	**<0.001**	**0.87**	**<0.001**
	(trivial)		**(small)**		**(moderate)**	
Initial collision vs. ground contact	−0.01	0.945	0.35	0.002	**0.46**	**<0.001**
	(trivial)		(small)		**(small)**	
Initial collision vs. play the ball	0.21	0.031	**0.52**	**<0.001**	**1.06**	**<0.001**
	(small)		**(small)**		**(moderate)**	
Secondary contact vs. ground contact	−0.2	0.068	−0.05	0.659	**−0.41**	**<0.001**
	(small)		(trivial)		**(small)**	
Secondary contact vs. play the ball	0.01	0.901	0.11	0.346	0.19	0.069
	(trivial)		(trivial)		(trivial)	
Ground contact vs. play the ball	0.21	0.078	0.17	0.223	**0.6**	**<0.001**
	(small)		(trivial)		**(moderate)**	
	**Ball carrier vs. tackler comparison for each tackle stage**					
Initial collision (*ball carrier* vs. *tackler*)	0.13	0.116	0.09	0.311	0.01	0.947
	(trivial)		(trivial)		(trivial)	
Secondary contact (*ball carrier* vs. *tackler*)	0.02	0.845	0	0.966	−0.1	0.343
	(trivial)		(trivial)		(trivial)	
Ground contact (*ball carrier* vs. *tackler*)	0.19	0.143	0.16	0.293	**0.44**	**<0.001**
	(trivial)		(trivial)		**(small)**	
Play the ball (*ball carrier* vs. *tackler*)	0.19	0.127	0.15	0.296	0.27	0.028
	(trivial)		(trivial)		(small)	
	**Head loading type**					
Direct vs. indirect	**0.35**	**<0.001**	0.16	0.004	**0.62**	**<0.001**
	**(small)**		(trivial)		**(moderate)**	

## Data Availability

Anonymised data available subject to reasonable request.
